# A simple respiratory severity score that may be used in evaluation of acute respiratory infection

**DOI:** 10.1186/s13104-016-1899-4

**Published:** 2016-02-12

**Authors:** Hector Rodriguez, Tina V. Hartert, Tebeb Gebretsadik, Kecia N. Carroll, Emma K. Larkin

**Affiliations:** Vanderbilt Center for Asthma Research, Vanderbilt University, 1215 21st Avenue South 6000, Medical Center East, North Tower, Nashville, TN 37232-8300 USA

**Keywords:** Pediatrics, Respiratory infections, Pulmonary

## Abstract

**Background:**

Acute respiratory infections are ubiquitous and may have long-term implications on respiratory health. There are many scoring systems used to objectively measure severity of respiratory infections in clinical and research settings. A respiratory severity score derived exclusively from physical exam components (RSS-HR) was studied as an objective measure of disease severity and was compared to a previously described score that uses pulse oximetry as a component of its score (RSS-SO).

**Findings:**

A score was derived from 497 infants. The RSS-HR median score was higher in infants that were hospitalized (8.0) versus outpatient (4.0, p < 0.001), and those with lower respiratory tract infections (LRTI) (6.5) versus upper respiratory infections (URI) (1.0, p < 0.001). When discriminating upper versus LRTIs the concordance index of regression for RSS-HR was 0.91 and RSS-SO was 0.93.

**Conclusions:**

RSS-HR distinguishes disease severity based on level of care, as well as LRTI versus URI.

## Background

Acute respiratory infections (ARIs), particularly lower respiratory tract infections (LRTI), are the most common reason for hospitalization in infants [1]. Similarly, in the outpatient setting, ARIs are a common reason for ambulatory visits [2]. The importance of respiratory viral infections extend beyond acute illness and associated management, as it has been demonstrated that infections increase the risk of recurrent wheeze in older children, and the incidence of allergic asthma throughout childhood and into late adolescence [3, 4].

Considering the prevalence of ARIs, the use of an objective measure of respiratory illness severity would have implications in clinical management, as well as clinical research. There have been many LRTI severity scores that have been developed for various purposes, such as for the assessment of infant breathing in response to therapies, observer agreement of lower respiratory disease in infants, and predicting likelihood of positive pressure ventilation in infants with LRTI [5–8]. The majority of these scores are applied to hospitalized children where many healthcare resources and broader testing may be readily available. A simple respiratory severity score that has been demonstrated to have good interrater reliability is the modified Tal. This score ranges from 0 to 12, with a higher score indicating more severe disease. Each score is an aggregate of assigned values ranging from 0 to 3 in categories of respiratory rate, retractions, wheezing, and oxygen saturation in room air [9]. While three of these four variables are obtained through physical examination, there may be situations where pulse oximetry may not be readily available depending on the available health care resources and setting of care. It was hypothesized that using infant heart rate as part of a respiratory severity score (RSS-HR) could reliably be used as an alternative for oxygen saturation (RSS-SO) in the development of a modified Tal and could also be used to evaluate respiratory infection severity outcome measures in comparison with the RSS-SO.

## Methods

The Tennessee Children’s Respiratory Initiative (TCRI) was used to assess the RSS-HR and RSS-SO. TCRI is a prospective cohort of 674 term healthy children who were enrolled upon infant presentation with acute respiratory illness. The level of acuity [provider visit, emergency department (ED), or inpatient] was categorized into the highest level of care based on chart review at completion of care for the illness. Enrollment occurred during four winter respiratory viral illness seasons between 2004 and 2008. Details of the TCRI cohort have been previously published [10]. Four hundred and ninety-seven infants had a documented heart rate during acute illness. The two severity scores, and their association with clinical outcomes were assessed among these 497 infants.

A severity score (RSS-HR) was calculated for each infant based on chart extraction of heart rate value during the acute illness. The scores were obtained retrospectively and not used in clinical decision-making. If a patient had numerous vital signs documented, which was encountered for inpatient admissions, the most severe values noted in the first 24 h of admission were used to determine the composite score. If patients had numerous outpatient or ED visits around the enrollment date, vitals were only extracted from enrollment date. Values were determined based on the Tal respiratory score, with heart rate ranges based on values described by Destino et al. [9, 11]. Scoring is detailed in Table 1. LRTI and URI were defined using both the physician discharge diagnosis, and post-discharge chart review. A panel of pediatricians reviewed cases that were not clearly identified as either LRTI or URI and determined whether the illness represented an LRTI, URI, croup or “other” [10]. This study was approved by the Vanderbilt IRB.

**Table 1:** Respiratory severity scoring rubric

Score	Respiratory rate	Wheeze	Heart rate^a^	${\text{SpO}}_{2}^{\text{b}}$	Accessory muscle use
0	<30	None	<150	>95	None
1	30–45	End-expiratory only	151–160	94–95	Mild intercostal retractions
2	46–60	Entire expiration and inspiration with stethoscope	161–170	90–93	Moderate retractions
3	>60	Entire expiration and inspiration without stethoscope	>170	<90	Moderate retractions + head bobbing or tracheal tugging

^a^RSS-HR = respiratory rate + wheeze + heart rate + accessory muscle use

^b^RSS-SO = respiratory rate + wheeze + SpO_2_ + accessory muscle use

### Statistical analysis

Patients’ demographics were described as indicated using median and interquartile ranges, mean and standard deviation, or frequency and proportion. Cronbach’s alpha was used to analyze interrater reliability of the scores. Both RSS-SO and RSS-HR, were assessed for criterion validity using objective respiratory infection outcomes such the diagnosis type (LRTI vs. URI), or location of acute care (outpatient visit, ED, hospitalization).

## Results

Among the 497 infants, the majority (59 %) were white, 55 % were male, and the mean age of infants at enrollment was 16 weeks. The majority of subjects were inpatient, 83 % (n = 413) while 17 % (n = 84) were outpatient. Details of subject demographics are detailed in Table 2. A diagnosis of LRTI was made in 420 (85 %) of these patients, while 77 (15 %) were diagnosed with URI.

**Table 2:** Cohort demographics

Infant characteristics	
Gestational age, weeks (average ± SD)	38.9 ± 1.3
Infant age, weeks (average ± SD)	16 ± 14
Sex, N (%)	
Male	275 (55 %)
Female	222 (45 %)
Infant race, N (%)	
Black	92 (19 %)
White	287 (58 %)
Hispanic	64 (13 %)
Other	53 (11 %)
Not answered	1 (0 %)
Insurance, N (%)^a^	
None	34 (7 %)
Private	148 (30 %)
Medicaid	314 (63 %)
Inpatient versus outpatient^b^, N (%)	
Inpatient	413 (83 %)
Outpatient	84 (17 %)

^a^Data missing for one infant

^b^Emergency room visits and outpatient visits were collapsed into the category of outpatient visits

**Fig. 1:**
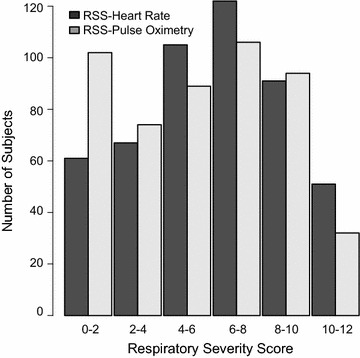
Score distribution comparison for respiratory severity score (RSS) with heart rate or with pulse oximetry. Side-by-side *bar graphs* comparing the number of subjects that fall within each scoring range using RSS-heart rate (*black*) or RSS-pulse oximetry (*gray*)

The median RSS-HR of inpatient enrollees was 8.0 [IQR: 6, 9], while outpatient was 4.0 [IQR: 2, 5.5] (p < 0.001). Similarly RSS-SO scores were higher for inpatients, 7.0 [IQR: 4, 9], while outpatient scores were 1.0 [IQR: 1, 4] (p < 0.001). The median RSS-HR in patients with LRTI was a median of 8.0 [IQR: 6, 9.5], while those with URI had a median score of 4.0 [IQR: 2, 5] (p < 0.001). Using the RSS-SO, the median score for LRTI was 6.5 [IQR: 4, 9], while for URI was 1.0. [IQR: 1, 2] (p < 0.001). Figure 1 compares the distribution of both scores. While both scores seemed to be reflective of disease severity, the internal consistency was better with scores based on pulse oximetry when compared to heart rate, with a Cronbach’s α of 0.76 versus 0.66 respectively.

We compared the performance of the two scores in discriminating outcomes of URI versus LRTI with the assessment metric of concordance index (C-index). Using a multivariable logistic regression that adjusted for infant age, gender and race, the C-index of the regression model for RSS-SO 0.93 (95 % CI 0.91, 0.96) and for RSS-HR C index was 0.91 (0.88, 0.94). Additional adjustment for fever in assessing criterion validity made no difference in predicting URI versus LRTI.

## Discussion

Our study demonstrated that it may be possible to broaden the applicability of the modified Tal by using an alternative score replacing the factor of oxygen saturation with heart rate. The modified Tal has been demonstrated to be internally valid in LRTI, and demonstrated utility determining disease severity in ARI [9, 12]. The RSS-HR was significantly higher in children diagnosed with LRTI compared to those with URI. In addition, it was significantly higher in those admitted to the hospital compared to those managed as outpatient. These findings may have clinical implications, and usefulness in research as it could be used to not only distinguish URI versus LRTI, but also determine the level of acuity of respiratory illness. This usefulness may be especially true in locations where assessment tools like pulse oximetry are not readily available, such as underserved communities and developing/undeveloped countries.

There were limitations to our study. When using heart rate, there was less interrater reliability. This may be explained by the fact that results were extracted from medical records in various levels of medical care and heart rate was obtained from different caregivers who may obtain heart rate by different means. Other factors that influence heart rate, like pain or agitation, were not accounted for in this study. The patients enrolled were at a single academic institution that is a referral center for numerous local hospitals, and as such, may possibly be more ill than those at other hospitals. The majority enrolled were also inpatient, which could further skew results towards a more severe population and influence scores measured. We also counted the highest heart rate and other vital signs, which may bias results as patients who are admitted have more data points to observe compared to patients presenting as outpatient who may not present with the most severe set of vitals. This would potentially skew subjects enrolled in the inpatient setting to having higher respiratory severity scores.

While acknowledging these limitations, we have described an additional tool to measure illness severity in ARI. This score has the advantage of being ascertained exclusively through physical exam, without the need for additional equipment. It is possible that this objective measure can have applications in both the clinical management of children presenting with viral respiratory illness, and in clinical research as it strongly associates with disease severity and clinical level of care. Future directions for the RSS-HR could include blinded, prospective use of the score and the potential associations with inpatient admission and LRTI versus URI.

## Abbreviations

ARI: acute respiratory illness
RSS: respiratory severity score
RSS-HR: respiratory severity score derived from heart rate
LRTI: lower respiratory tract infection
URI: upper respiratory tract infection
CI: confidence interval
IQR: interquartile range
